# Polygenic risk prediction based on singular value decomposition with applications to alcohol use disorder

**DOI:** 10.1186/s12859-022-04566-5

**Published:** 2022-01-10

**Authors:** James J. Yang, Xi Luo, Elisa M. Trucco, Anne Buu

**Affiliations:** 1 Department of Biostatistics and Data Science, University of Texas Health Science Center, Houston, USA; 2 Department of Psychology, Florida International University, Miami, USA; 3 Department of Psychiatry, University of Michigan, Ann Arbor, USA; 4 Department of Health Promotion and Behavioral Sciences, University of Texas Health Science Center, Houston, USA

**Keywords:** Polygenic risk score, Singular value decomposition, Complex disease, Alcohol use disorder

## Abstract

**Background/aim:**

The polygenic risk score (PRS) shows promise as a potentially effective approach to summarize genetic risk for complex diseases such as alcohol use disorder that is influenced by a combination of multiple variants, each of which has a very small effect. Yet, conventional PRS methods tend to over-adjust confounding factors in the discovery sample and thus have low power to predict the phenotype in the target sample. This study aims to address this important methodological issue.

**Methods:**

This study proposed a new method to construct PRS by (1) approximating the polygenic model using a few principal components selected based on eigen-correlation in the discovery data; and (2) conducting principal component projection on the target data. Secondary data analysis was conducted on two large scale databases: the Study of Addiction: Genetics and Environment (SAGE; discovery data) and the National Longitudinal Study of Adolescent to Adult Health (Add Health; target data) to compare performance of the conventional and proposed methods.

**Result and conclusion:**

The results show that the proposed method has higher prediction power and can handle participants from different ancestry backgrounds. We also provide practical recommendations for setting the linkage disequilibrium (LD) and *p* value thresholds.

## Introduction

Genome-wide association studies (GWAS) have been used to identify variants that are significantly associated with the phenotype of interest. Yet, for complex diseases such as substance use disorders (SUD), the phenotype tends to be influenced by a combination of multiple genes or variants, each of which has a very small effect. As a result, many GWAS with small to moderate sample sizes fail to identify important variants even though the phenotype has been shown to be highly heritable. This phenomenon is called the “missing heritability problem” [1]. Although increasing the sample size of GWAS or conducting a meta analysis on several studies are possible remedies to reach sufficient statistical power, they may not be feasible in some practical settings. An alternative approach that shows promise is the polygenic risk score (PRS), also known as genetic risk score or risk profile score [2]. PRS derived its name from the notion that complex diseases are highly polygenic [3] with the effect of each variant being very small. To deal with this issue, the PRS approach proposes an additive model to summarize the marginal effects of many variants to quantify genetic influences on a particular phenotype [4]. Thus, PRS represents the distribution of aggregated genetic liability that can be used to profile the genetic contribution to the phenotype. To our knowledge, Wray et al. [5] was the first study to apply the PRS approach in GWAS.

PRS has been applied to therapeutic intervention, disease screening, and life planning [6]. For example, PRS was used to predict onset and early patterns of heavy episodic drinking in males [7]. The well-known Adolescent Brain Cognitive Development Study (https://abcdstudy.org/) also demonstrated its ability to predict cognitive performance in a large sample of 9–10 years old children in the US population [8]. Further, PRS was integrated with family history and traditional risk factors to improve the screening for coronary heart disease [9].

In spite of the above potential applications of PRS, how to accurately estimate PRS remains an open research question. Because the allele frequencies of variants and the linkage disequilibrium (LD) patterns vary across different populations [10], constructing PRS without considering these two key factors is likely to result in either bias or lower power. According to a recent comprehensive review of existing PRS studies [11], 67% of studies included exclusively European ancestry participants; 19% included only East Asian ancestry participants; and only 3.8% were among cohorts of African, Hispanic, or Indigenous individuals. Importantly, the same study showed that the predictive performance of European ancestry-derived PRS is lower in non-European ancestry samples with the worst performance found among African ancestry samples. This is the so-called transferability issue with PRS [12].

In this paper, we review conventional PRS methods and identify important methodological issues. To deal with these issues, we propose a PRS method based on lower rank approximation of the observed genotypes and eigen-correlation selection. Empirical data collected from the substance use field are chosen to demonstrate the applications of these PRS methods because substance use disorders (SUD) are highly heritable [13–15] and many variants have been identified to be associated with SUD [16]. Secondary data analysis is conducted to compare different PRS methods in terms of their performance. The results also shed some light on future applications of these methods.

## Review of conventional PRS methods

In general, the PRS method requires two independent data sets: the discovery data and the target data. The discovery data is used to identify the set of variants associated with the phenotype and estimate their effects. These estimated effects are later applied to the genotypes of the participants in the target data to calculate their PRS.

Let ${\hat{\beta }}_j$ be the marginal effect size for Variant *j* ($j=1,\ldots m$) estimated from the discovery data; and $g_{ij}$ be the genotype coded as the number of the effect allele at Variant *j* for Individual *i* from the target data. The PRS for Individual *i* is calculated as

1 $$\begin{aligned} S_i=\sum _{j=1}^m {\hat{\beta }}_j g_{ij}. \end{aligned}$$

Equation (1) indicates that the PRS is an additive function of the genotype $g_{ij}$. The PRS for Individual *i* in the target data is the weighted sum of his/her genotype $g_{ij}$ with the weights ${\hat{\beta }}_j$ estimated from the discovery data.

Based on Eq. (1), the performance of PRS depends on the set of variants $g_{ij}$ and the effect size of each variant ${\hat{\beta }}_j$. In a typical setting of GWAS, the number of variants well exceeds 1 million whereas the number of participants is usually between 1000 and 10,000. If all the variants are included for calculating PRS, it is not feasible to jointly model all variants and estimate their effects accurately. In fact, the majority of variants are not likely to be associated with the phenotype.

Choi et al. [17] summarized various approaches for PRS construction. Among them, the clumping and thresholding approach is widely used because of its simplicity and relatively good performance. In addition, this approach only requires summary statistics rather than the original genetic data which are usually not publicly accessible due to confidentiality issues. The clumping step identifies the variant with the strongest association with the phenotype and removes neighboring variants that are in linkage disequilibrium with it. Thus, it produces a subset of variants which are in linkage equilibrium with one another. The thresholding step further reduces the number of variants identified in the clumping step by only keeping those variants if their *p* values are smaller than a given threshold. The optimal values of clumping and thresholding are usually determined by a model selection procedure. The details of these steps can be found in Choi et al. [17] and this approach was adopted by the popular PRS software: PRSice [18]. Another popular approach—based on a Bayesian model—takes the linkage disequilibrium among variants into account and models the faction of causal variants in the prior distribution. The Markov chain Monte Carlo method is used to estimate the shrink effects of variants. This approach was implemented in LDpred [19] and its improved version: LDpred2 [20].

## Important methodological issues of PRS

The performance of PRS depends on not only the chosen variants but also the quality of estimates of their effects. The traditional PRS construction usually follows the method described in Purcell et al. [21]. The fist step is to separate discovery samples into ancestrally homogeneous subgroups. The next step is to derive principal components such as 10 in each group and add these 10 principal components as covariates in the regression model to estimate the effect of each variant. Some researchers further adjusted for additional covariates such as age and gender. The reason is that these leading principal components are confounded with population stratification or cryptic relatedness. Adding these covariates could correct for these effects so the adjusted effect of each variant may not be biased.

Yet, the $R^2$ value of the resultant PRS following this practice is usually only around 0.64–1.1% [7] in alcohol use behaviors, although in neuropsychiatric diseases up to 5–6% has been reported [22]. In fact, recent studies have shown the range of $R^2$ to be 0.5–3% [23–26]. These relatively small $R^2$ values raise a legitimate concern that the estimation of marginal effects of variants may not adequately reflect the polygenic contribution to the phenotypes. Specifically, the estimation may have been over-adjusted. For example, if the principal components derived from the genotypes of the discovery sample are highly correlated with race and ethnicity that happens to be a strong predictor of the phenotype, adjusting for the principal components would eliminate not only the effect of ethnicity but also the power to predict the phenotype using the adjusted variant effects. Furthermore, because the number of variants is much larger than the sample size, including more variants does not necessarily increase the prediction accuracy of the PRS estimate [27]. Thus, how to choose an informative subset of variants is critical. The present study proposes a new PRS method to deal with these issues.

## The proposed PRS method based on principal component projection

### Polygenic model

Suppose the discovery genotype data are organized as a $n\times m$ matrix *A* with each row corresponding to an individual and each column to a variant. Thus, the cell $g_{ij} (\in A)$ represents the genotype of Individual *i* on Variant *j*. For SNP data, $g_{ij}$ is coded as 0, 1, or 2 to reflect the number of the effect allele. Before calculating the principal components, $g_{ij}$ is normalized as $(g_{ij}- 2p_j)/\sqrt{2p_j(1-p_j)}$, where $p_j$ is the allele frequency of each variant [28]. Missing values are imputed with 0. The resulting normalized matrix of *A* is denoted by *Z*.

The effects of genotypes on the phenotypes of *n* subjecs $\varvec{y} = (y_1,\ldots ,y_n)^T$ can be characterized using the following linear random effect model:

2 $$\begin{aligned} \varvec{y} = \mu + Z\varvec{b} + {\varvec{e}} \end{aligned}$$

where $\mu$ is the intercept, *Z* is the normalized matrix of genotypes, $\varvec{b} \sim N(0,\sigma _a^2 I)$ is the random effect, and ${\varvec{e}} \sim N(0,\sigma _e^2 I)$ is the error. The genetic similarity matrix (or genetic relatedness matrix) among the *n* individuals is defined as $K= ZZ^T/m$. Equation (2) can then be written as

3 $$\begin{aligned} \varvec{y} = \mu + {\varvec{g}} + {\varvec{e}} \end{aligned}$$

where ${\varvec{g}} \sim N(0,\sigma _g^2K)$; and $\sigma _g^2 = m\sigma ^2_a$ is the variance of all the additive genetic effects. When the matrix *K* is known or can be derived from the pedigree of the *n* individuals, we can estimate the parameters in Eq. (2) or (3) based on the genotypes and phenotypes. However, when the matrix *K* needs to be estimated from the genotypes, the number of unknown parameters is larger than the number of data points so the proposed linear random effect model ends up overfitting the data [29]. Therefore, when the matrix *K* needs to be estimated from the same data, the estimates of parameters in Eq. (3) are biased.

Using the singular value decomposition (SVD), the matrix *Z* can be expressed as $Z=U\Lambda V^T$, where *U* and *V* are both orthogonal matrices and $\Lambda$ is a rectangular diagonal matrix with non-negative singular values $(\lambda _1,\lambda _2,\ldots )$ on the diagonal. In practice, we rearrange the column vectors of *U*, *V*, and $\lambda$’s so that $\lambda _1 \ge \lambda _2\ge \ldots$. Following the convention of GWAS analysis, we define the principal components of *Z* as the column vectors of the left singular matrix *U* and the eigenvalues of *Z* as the square of singular values of *Z*. Equation (2) can thus be written as

$$\begin{aligned} \varvec{y}&= \mu + U\Lambda V^T\varvec{b}+{\varvec{e}} \end{aligned}$$

If we define $\beta = \Lambda V^T\varvec{b}$, then we have

4 $$\begin{aligned} \varvec{y}= \mu + U \beta + {\varvec{e}}. \end{aligned}$$

Hence, the linear random effects model (Eq. 3) can be written as a linear regression model on the principal components *U* (Eq. 4). In addition, modeling all the principal components in Eq. (4) as either random or fixed effects shares the same underlying regression model [30].

To address the issue of more parameters than data points in Eq. (4), we propose to use a subset of principal components in the regression model to approximate the full model as follows:

5 $$\begin{aligned} \varvec{y}= \mu + \sum _{k \in S}u_k\gamma _k +{\varvec{e}} \end{aligned}$$

The details about how to select the indexes in the set *S* and the variants used to estimate $u_k$ are described in the following sections. We also demonstrate how to estimate the parameters in Eq. (5) based on the discovery data and apply them to the target data.

### Variant selection

The SVD of the genotype matrix *Z* does not require information about the phenotypes. Since we propose to use the principal components (i.e., the left singular vectors) as predictors of the phenotypes, choosing variants significantly associated with the phenotypes and using these variants to derive the principal components would increase the association in Eq (5). For this reason, we propose a two-step approach to select variants based on the marginal *p* value of each variant’s linear association with the phenotype via a simple linear regression. The first step, LD-based clumping, selects the most significant variant in a region and removes other variants in the same region that are in LD with the the chosen variant. This process is repeated for all regions. After clumping, the final set of variants are in approximate linkage equilibrium with each other. The above procedure is carried out by the plink program with command options –clump-kb 500–clump-p1 1 –clump-r2 $\rho$ (where $\rho$ is the LD threshold) so that variants within 500 kb are in linkage equilibrium after clumping. In the second step, a subset of variants is chosen if their *p* values are smaller than the threshold $\theta$. In this study, we evaluate different values of $\rho$ and $\theta$ in terms of the prediction power of PRS (see details in the results section).

### Principal component selection

Once the variants are selected, the next critical step is to determine the number of principal components (left singular vectors) used in Eq. (5). The minimum requirement for a model to be estimable is that the number of principal components is smaller than the sample size. However, too many principal components may increase the variance in the estimates. The common practice is to choose the number based on a fixed number (e.g., 10) or based on the eigenvalues greater than a fixed threshold. These methods, however, do not consider the correlation between each principal component and phenotype. We propose an alternative approach that takes this into account.

Define eigen-correlation (EigenCorr) as the correlation between a principal component ($u_k$) and the phenotype ($\varvec{y}$) multiplied by the corresponding singular value ($\lambda _k$): $\text {EigenCorr}_k=cor(u_k, \varvec{y})\lambda _k.$ Lee et al. [31] showed that the sum of all squared correlations between each variant and the phenotype is equal to the sum of all squared EigenCorr’s. Since the majority of principal components are uncorrelated with the phenotype, $\text {cor}(u_k,\varvec{y})$ approximately follows $t/\sqrt{n-2+t^2}$ where *t* is a *t*-distribution with $n-2$ degrees of freedom. In PRS studies, the sample size *n* is usually 1000 or larger in the training data so the 95% confidence interval for $\text {cor}(u_k,\varvec{y})$ is within $(-0.062, 0.062)$ when the principal component and the phenotype are uncorrelated. Among singular values calculated from the normalized genotype matrix, the largest singular value depends on *n* and *m*. We simulated GWAS data with a common setting of $n=1000$ and $m =100,000$ and found that the largest singular value is less than 5 and the majority of values are around 1 or smaller. Thus, the square of eigen-correlations are less than 0.1 ($0.062^2\times 5^2$) for most principal components. Based on these results, we propose to select the indexes in the set of *S* in Eq. (5) when their corresponding squared EigenCorr is above 0.1.

### Principal component projection

The SVD of *Z* is $Z=U\Lambda V^T$ where *Z* is an $n\times m$ matrix. When *m* is larger than *n*, a direct calculation of SVD is time consuming. However, if the purpose is to find the first few columns of *U* matrix, we can first calculate $\Phi = ZZ^T$ and then use the spectral decomposition on $\Phi$ to calculate its eigenvectors $(u_1,u_2,\ldots )$ and eigenvalues $(\sigma _1,\sigma _2,\ldots )$, where $\sigma _1 \ge \sigma _2\ge \ldots$. Given these eigenvectors and eigenvalues, the left singular matrix is $U=(u_1,u_2,\ldots )$ and the singular values of *Z* are $\lambda _k = \sqrt{\sigma _k}$ $(k=1,2,\ldots )$. Thus, the right singular matrix $V=(v_1,v_2,\ldots )$ can be derived as:

6 $$\begin{aligned} v_k=Z^Tu_k/\lambda _k \end{aligned}$$

for $$k=1,2,\ldots$$.

Equation (6) can be written, equivalently, as $u_k=Z v_k/\lambda _k$, which indicates that the eigenvector $u_k$ can be derived from the discovery data *Z* by projecting *Z* through $v_k$ and weighting it by $1/\lambda _k$. Following this idea, we propose to derive the corresponding eigenvector in the target genotype data, say *B*, by projecting *B* through $v_k$ and $\lambda _k$ (both are calculated from *Z*) as follows:

7 $$\begin{aligned} u_k^{(B)} = B v_k/\lambda _k, \end{aligned}$$

### PRS construction

The eigenvector $u_k$ derived from the discovery genotype data *Z* is used to estimate the effect size of the principal component, whereas the corresponding eigenvector in the target genotype data *B*, $u_k^{(B)}$, is employed to construct the PRS. Specifically, given $\varvec{y}$ and $u_k$ from the discovery data *A*, we can use Eq. (5) to derive the least squared estimates ${\hat{\gamma }}_k$, which is then used as the effect size to calculate the PRS for the subject in the target sample as:

8 $$\begin{aligned} \text {PRS} = \sum _{k\in S} {\hat{\gamma }}_{k} u_{k}^{(B)} \end{aligned}$$

## Secondary data analysis

### Databases

In this study, we used three sources of genomic data to demonstrate the applications of the proposed PRS method and evaluate its performance relative to the conventional method. The 1000 Genome Project Phase 3 reference panel was used as our reference genomic data. This publicly accessible database (http://bioinfo.hpc.cam.ac.uk/downloads/datasets/vcf/index_.html) contains genetic data from 2504 individuals who were classified based on 5 super populations: African (AFR), Ad Mixed American (AMR), East Asian (EAS), European (EUR), and South Asian (SAS). We used these five super populations to represent 5 distinct genetic ancestries.

The discovery (training) database was distributed by the Study of Addiction: Genetics and Environment (SAGE) (dbGaP study accession: phs000092.v1.p1; https://www.ncbi.nlm.nih.gov/projects/gap/cgi-bin/analysis.cgi?study_id=phs000092.v1.p1). The SAGE aggregated data containing common measures from three large scale studies in the substance abuse field: the Collaborative Study on the Genetics of Alcoholism (COGA), the Family Study of Cocaine Dependence (FSCD), and the Collaborative Genetic Study of Nicotine Dependence (COGEND). There were 4094 participants in this database.

The target (testing) database came from the National Longitudinal Study of Adolescent to Adult Health (Add Health) (dbGaP study accession: phs001367.v1.p1; https://www.ncbi.nlm.nih.gov/projects/gap/cgi-bin/study.cgi?study_id=phs001367.v1.p1). The Add Health (Harris et al. 2013) collected GWAS data and health behavior data from a large sample of U.S. adolescents who were followed from grades 7–12 into adulthood. Genetic data were available for 9974 participants with the primary race groups being Black and White.

### Imputation

The genomic data from 1000 Genome Project, SAGE, and Add Health were genotyped from different types of SNP genotype arrays. The genomic data from Add Health have been imputed, whereas the SAGE did not provide imputed data. To ensure that all the three databases cover the same variants, we conducted imputation on the SAGE data using the imputation service provided by the Michigan Imputation Center (https://imputationserver.readthedocs.io/en/latest/).

While the genomic data of 1000 Genome Project and Add Health were both based on GRCH37/hg19, the genomic data of SAGE were based on NCBI Build 36.1. Because the Michigan Imputation Center can only impute genomic data built on GRCh37/hg19 or GRCh38/hg38, we first converted the genome coordinate of the SAGE data to GRCH37/hg19 genomic build using the liftover program [32]. A quality control procedure (removing variants with the MAF $< 0.01$, the *p* value of Hardy–Weinberg Equilibrium test $<10^{-6}$, the missing rate $< 0.05$) was also conducted before the imputation. We chose the Eagle v2.4 for phasing and the Minimac4 for imputation with the reference panel for both procedures being the 1000 Genomes Phase 3 (Version 5). After the imputation, we conducted further quality control by keeping those variants with the imputation R-square value being greater than 0.3 for data analysis.

### Participant selections

After the imputation, we extracted the common variants across the three data sources for data analysis. In the SAGE and Add Health databases, we focused the analysis on those participants with the majority of genomic compositions being either AFR or EUR ancestry because the sample sizes of participants with other ancestries were very small in both studies.

We used the ethnic information of the 1000 Genome participants to infer the ancestries of the SAGE and Add Health subjects. We first merged the three databases and then conducted principal components analysis on the merged data using the PLINK software [33, 34]. A Fisher linear discriminant function was built by using the top twenty principal components in the 1000 Genome data as predictors and their ethnicity as outcomes. Applying this Fisher linear discriminant function to the SAGE and Add Health data, we were able to calculate the posterior probabilities corresponding to the five ancestry groups (i.e., the five super-populations defined by the 1000 Genome Project). The participants in SAGE and Add Health were then chosen if their posterior probabilities in either AFR or EUR were above 0.9. This process identified 3394 SAGE participants and 8588 Add Health participants.

### Quality control for calculating PRS

Although both the SAGE and Add Health genomic data were imputed using the 1000 Genome Project reference panel, it is still necessary to eliminate the ambiguous SNPs which have complementary alleles (either A/T or C/G). This is a recommended quality control procedure for PRS as it eliminates the potential canceled effects when comparing the proposed method and the conventional method. After the removal, the three databases shared 2,993,682 variants in common.

### Phenotype selection

Both the SAGE and Add Health studies measured many substance use related phenotypes. In this study, we focused on the number of lifetime alcohol use disorder symptoms because both studies adopted the DSM IV criteria. SAGE provided the number of alcohol dependence symptoms (0–7), whereas Add Health measured the number of alcohol use disorder symptoms (0–11).

### Method comparison

We applied the following three PRS methods to analyze the imputed data of SAGE (discovery) and Add Health (target):

*The conventional method*: PRS $=\sum _{j\in S_1} {\hat{\beta }}_j g_{j}$
*The Bayesian method*: LDpred2
*The proposed method*: PRS $=\sum _{k\in S_2} {\hat{\gamma }}_{k} u_{k}^{(B)}$

The conventional method summed up the effects of variants selected in the discovery data ($j \in S_1$), with the effect size for each variant (${\hat{\beta }}_j$) being derived from marginal regression with 10 principal components as covariates. The LDpred2 method was implemented in the bigsnpr package (https://github.com/privefl/bigsnpr). The proposed method was described in details in “The proposed PRS method based on principal component projection” section. The former two methods were chosen for method comparison because (1) the conventional method which was based on clumping and thresholding of *p* values was relatively straightforward and yet performed comparably to other existing PRS methods [17]; and (2) the LDpred2 method represented a newer alternative method based on the Bayesian paradigm. Both are popular PRS methods.

## Results

We used the method described in “Participant selections” section to identify ancestrally homogeneous subgroups of AFR and EUR for both SAGE and Add Health datasets using the 1000 Genome Project as the reference genomic data. In the SAGE dataset, 1308 AFR and 2675 EUR were identified, whereas in the Add Health dataset, 1362 AFR and 3959 EUR were found. The analysis was restricted for these participants in order to evaluate the PRS for either ancestrally homogeneous or diverse groups.

**Table 1:** Summary statistics of alcohol phenotypes in SAGE and Add Health

	AFR	EUR	*p* value
SAGE	$n=$ 1308	$n=$ 2675	
Alcohol dependence symptoms	2.88 (2.55)	2.77 (2.55)	0.188
Add Health	$n=$ 1362	$n=$ 3959	
Alcohol use disorder symptoms	0.80 (1.89)	2.10 (2.84)	< 0.001

The summary statistics (means and standard deviations) of the phenotype variables described in “Phenotype selection” section are shown in Table 1. The high average number of alcohol dependence symptoms in the SAGE dataset (about 3 out of 7) reflected the nature of high-risk samples. Conversely, the average number of alcohol use disorder (AUD) symptoms (0.80–2.10 out of 11) in the Add Health dataset was low because the sample represented the general population. The two-sample *t*-tests examining racial differences indicate that there was no significant difference between AFR and EUR participants in the SAGE study. However, in the Add Health study, EUR tended to have a higher level of AUD symptomatology than AFR. This is again consistent with prevalence data in the general population.

**Fig. 1:**
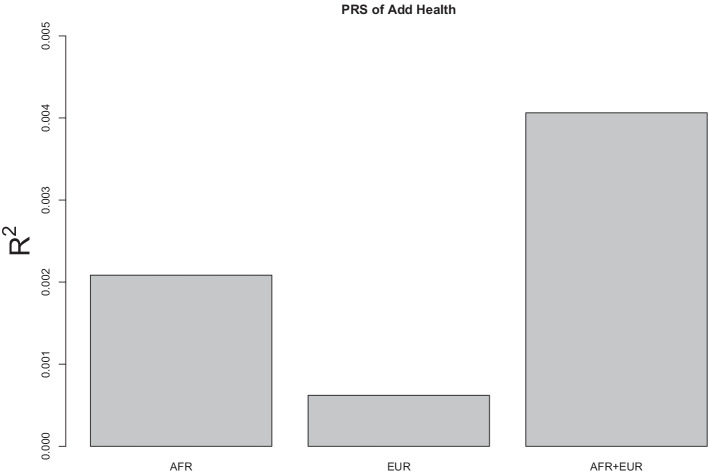
Barplots of $R^2$ values for predicting AUD symptoms of Add Health participants using the PRS calculated from SAGE discovery data based on the conventional method. AFR: conducted PRS on AFR ancestral group only. EUR: conducted PRS on EUR ancestral group only. AFR+EUR: conducted PRS on AFR and EUR ancestral groups

In this study, we constructed PRS for Add Health participants using SAGE participants as the discovery sample. Three PRS methods were applied to analyze the data: the conventional method, the Bayesian method, and the proposed method. For each method, the analysis was conducted on the AFR only, the EUR only, and the AFR and EUR together. Based on the conventional method, the effect size of each variant was estimated using linear regression with 10 principal components as covariates. The PRS was then constructed following the clumping and thresholding procedure with various clumping cut-off values ($r^2= 0.01, 0.1, 0.2$) and thresholding cut-off values (at 0.0001, 0.001, 0.01, 0.1, 0.2, 0.3, 0.4, 0.5, 1). The coefficient of determination ($R^2$) was calculated for each combination of cut-off values to indicate the proportion of variance in AUD symptoms explained by the PRS. This statistic was also used to evaluate the performance of PRS. Figure 1 shows the largest $R^2$ value (among all combinations of cut-off values) for AFR only, EUR only, and AFR+EUR, indicating that the conventional method performed poorly across the three samples (all $R^2$ values were less than 0.005).

**Fig. 2:**
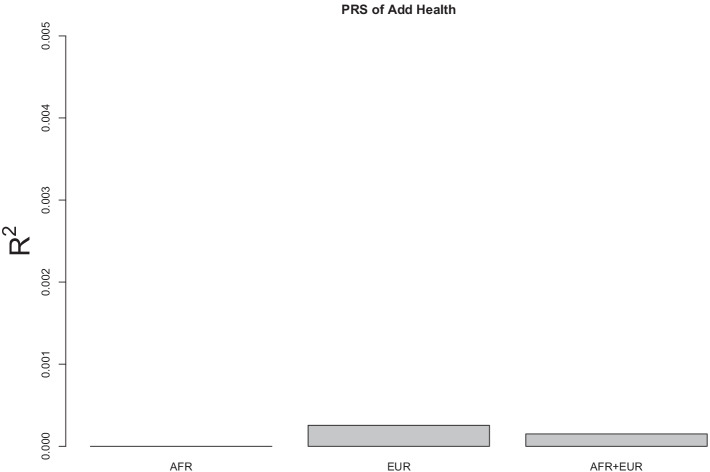
Barplots of $R^2$ values for predicting AUD symptoms of Add Health participants using the PRS calculated from SAGE discovery data based on the Bayesian method. AFR: conducted PRS on AFR ancestral group only. EUR: conducted PRS on EUR ancestral group only. AFR+EUR: conducted PRS on AFR and EUR ancestral groups.

The results using the Bayesian method, LDpred2, are shown in Fig. 2. All the $R^2$ values in AFR only, EUR only, and AFR+EUR were smaller than 0.001. In comparison to the conventional method (Fig. 1), this Bayesian method actually performed worse.

**Fig. 3:**
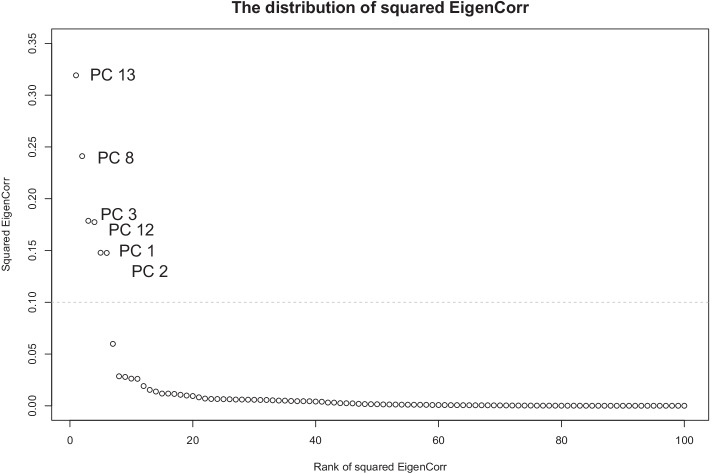
The scatter plot of the top 100 squared EigenCorr values versus their ranks. The dotted horizontal line is the cut-off value where the principal components with their corresponding EigenCorr above the dotted line are selected for PRS model construction. Each panel is based on a given $\rho$ value in the first step and $\theta$ value in the second step for variant selection used for SVD.

The proposed method was also used to conduct PRS analysis so the performance can be compared with that of the conventional method. An important step of the procedure is to calculate EigenCorr for identifying which of the principal components were more correlated with the phenotype. Based on $\rho =0.2$ to select variants in linkage equilibrium (i.e., the sample linkage correlation between each pair of variants is less than 0.2), we calculated SVD of the SAGE dataset and ranked the squared EigenCorr. The distribution of squared EigenCorr is presented in terms of its rank in Fig. 3, showing that the largest squared EigenCorr were derived from the 13th and 8th principal components. Although the first principal component had the largest eigenvalue, it was ranked 5th. In addition, based on the cut-off value of 0.1 (the dashed line), we identified six large EigenCorr’s corresponding to the 13th, 8th, 3rd, 12th, 1st, 2nd principal components, which were used for PRS construction. If we used eigenvalues to choose principal components, we would end up choosing only the 1st principal component (with the eigenvalue value of 159) because the 2nd (eigenvalue = 3.8), the 3rd (eigenvalue = 2.1), and the remaining principal components (all with eigenvalues being close to or less than 1) all had very small eigenvalues in comparison.

**Fig. 4:**
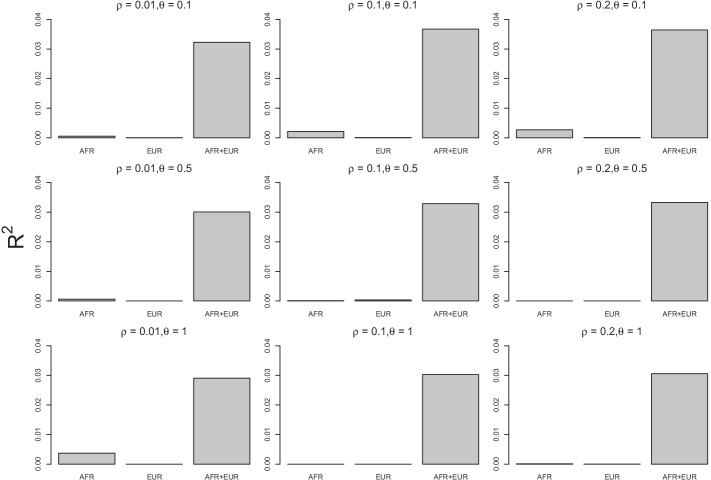
Barplots of $R^2$ values for predicting AUD symptoms of Add Health participants using the PRS calculated from SAGE discovery data based on the proposed method. AFR: conducted PRS on AFR ancestral group only. EUR: conducted PRS on EUR ancestral group only. AFR+EUR: conducted PRS on AFR and EUR ancestral groups.

We evaluated the performance of PRS based on the $R^2$ under different values of the LD threshold ($\rho =0.2, 0.1, 0.01$) and the *p*-value threshold ($\theta =0.1, 0.5, 1$). The results are shown in Fig. 4 using barplots. The $R^2$ was calculated for AFR only, EUR only, or the two combined. While all the $R^2$ values corresponding to AFR only and EUR only were smaller than 0.01, the $R^2$ values were above 0.03 across different values of $\rho$ and $\theta$ if we used participants from the mixture of AFR and EUR. This set of analyses also informs the choice of the values of $\rho$ and $\theta$. For large $\rho$ values, the selected variants are likely to be in linkage disequilibrium. On the other hand, for small $\rho$ values, we may eliminate variants that are informative. The value of $\rho$ at 0.1 is thus a good compromise. In terms of the $\theta$, we recommend to set it at 0.1 to increase information contents in driving principal components. Another advantage of choosing $\rho$ at 0.1 and $\theta$ at 0.1 is to reduce computational time during SVD and principal component projection because it involves a small number of variants. Under $\rho =0.1$ and $\theta =0.1$, the $R^2$ values for the AFR and EUR combined is 0.037, indicating that the proposed method can explain 3.7% of the variation in AUD symptoms with the PRS built upon the 2nd, 3rd, and 1st principal components.

## Discussion

Our proposed method did not attempt to fit the random effect model in Eq. (3) directly for the following reasons: (1) the estimates are likely to be inconsistent because the number of unknown parameters is larger than the sample size; (2) the procedure would be very time-consuming; and (3) how to apply the fitted model to the genotypes of participants in the target sample is an open research question. We, instead, proposed to fit Eq. (6) so it can be applied to the discovery data by projecting the observed genotypes to the axes of principal components. In this way, we have dealt with all the above issues.

Although the conventional PRS method can be implemented easily and it only requires the summary statistics of the discovery data instead of the original genotype data, it has a critical issue. Adjusting the first few principal components while deriving the effect of each variant is actually equivalent to estimating the variant effect from the remaining principal components. Although this procedure may adjust for the large structure effect, the derived effects of variants may still depend on other confounding factors such as demographic or socio-economic status [35]. In fact, adding large PCs in the regression model may over-adjust the estimates of marginal variant effects. Particularly, given the purpose of PRS is to build a *prediction model* for the phenotype based on genotypes, adding even 1 principal component as a covariate is expected to reduce the prediction accuracy [27].

Unlike the conventional method that only requires summary statistics from the discovery data, our approach requires availability of the singular values and right singular matrix of the discovery data. Nevertheless, users would not be able to recover the original genetic data based on these available information. Thus, the confidentiality of participants in the discovery data can still be kept. Moreover, although this study only dealt with two ancestry groups because they were the majority in the discovery and target samples, the proposed method can be easily applied to more ancestry groups.

## Conclusions

This study makes a unique contribution to the literature by proposing a new PRS method that has several strengths. First, the proposed method has higher prediction power than the conventional method that tends to commit over-adjustment during estimation of marginal effects of variants. Second, our approach based on principal components that are linear transformations from the genotype matrix conforms to the commonly accepted theory of additive genetic variance for complex traits [36]. Third, the principal components selected by the proposed method can facilitate our understanding of the structure of genetic effects on the phenotype. Fourth, our approach can handle participants from different ancestry backgrounds as long as the ancestries of participants in the target sample are a subset of those in the discovery sample.

## Data Availability

The Julia program will be deposited on GitHub at https://github.com/jjyang2019/SVD_Projection.jl

## Abbreviations

Add Health: National longitudinal study of adolescent to adult health
GWAS: Genome-wide association studies
LD: Linkage disequilibrium
PRS: Polygenic risk score
SAGE: Study of addiction: genetics and environment
SUD: Substance use disorders
SVD: Singular value decomposition
